# Wesselsbron virus at the livestock–human interface: A practical guide to disease recognition and diagnosis

**DOI:** 10.1016/j.onehlt.2026.101583

**Published:** 2026-09-18

**Authors:** Kelly Garina Mugisha, Obdulio García-Nicolás, Moses M. Njire, Cecilia R. Waruhiu, Charaf Benarafa, Eric M. Fèvre

**Affiliations:** a International Livestock Research Institute, PO Box 30709, Nairobi 00100, Kenya; b Jomo Kenyatta University of Agriculture and Technology, School of Biological Sciences, P.O. BOX 62000, City square, Nairobi, 00200, Kenya; c Institute of Virology and Immunology (IVI), Sensemattstrasse 293, Mittelhäusern 3147, Switzerland; d Department of Infectious Diseases and Pathobiology, Vetsuisse Faculty, University of Bern, Länggasstrasse 122, Bern 3012, Switzerland; e Multidisciplinary Center for Infectious Diseases, University of Bern, Bern, Switzerland; f The African Genomics Centre Consultancy, Nairobi Area, Nairobi 00100, Kenya; g Institute of Infection, Veterinary & Ecological Sciences, University of Liverpool, L69 3BX, UK.

**Keywords:** One Health, *Orthoflavivirus*, Pathogenesis, Diagnostic, Surveillance, Rift Valley fever, Differential diagnosis

## Abstract

Wesselsbron virus (WSLV) is a mosquito-borne *Orthoflavivirus* of the yellow fever virus (YFV) serocomplex, widely distributed across sub-Saharan Africa. It is neglected, and its true impact is likely underestimated. WSLV causes abortion, congenital malformations, and neonatal mortality in sheep, the primary recognized vertebrate host, but infections have also been documented in goats, cattle, camels, wildlife, and humans. The virus is maintained in complex cycles predominantly involving *Aedes* mosquitoes and incompletely defined domestic and wild vertebrate hosts. Direct infection via the placenta, milk, and accidental laboratory exposure have been documented. These non-vector routes coincide with routine husbandry in African pastoral and agropastoral systems, creating opportunities for exposure at the livestock-human interface. Severe clinical disease is best characterized in sheep, in which hepatitis, congenital abnormalities, abortion, and neonatal mortality predominate. Similar symptomatology was reported in camels, but causality has not yet been experimentally demonstrated. Human infections are sporadic, usually self-limiting, it can involve hepatic disease, and rarely neurological manifestations. Clinical diagnosis of Wesselsbron disease is challenging because symptoms and environmental circumstances overlap closely with Rift Valley fever. While there is no serological cross-reactivity between WSLV and RVFV, WSLV antibodies broadly cross-react with other viruses of the YFV serocomplex. Molecular assays have improved detection of acute infections, and recent experimental studies have clarified pathogenesis, transmission, and immune responses. This review provides a practical framework for recognizing WSLV infection, selecting appropriate diagnostic samples and assays, and deciding when WSLV should enter the differential diagnosis of Rift Valley fever-like disease in humans, ruminants, and camels. We highlight non-vector zoonotic transmission, improved surveillance, routine diagnostic inclusion, and targeted research to better define Wesselsbron disease in a One Health context.

## 1 Introduction

Wesselsbron virus (WSLV) is a neglected mosquito-borne arbovirus of the family *Flaviviridae*, genus *Orthoflavivirus* (formerly *Flavivirus*) [1]. *Orthoflaviviruses* are enveloped, positive-sense, single-stranded RNA viruses including yellow fever virus (YFV), dengue virus (DENV), Japanese encephalitis virus (JEV), West Nile virus (WNV), and Zika virus (ZIKV) [1], [2]. WSLV belongs to the YFV serocomplex and is phylogenetically closest to YFV [3]. First identified in 1955 from the liver of an aborted lamb in the Wesselsbron District of South Africa [4], WSLV has since been detected across Sub-Saharan Africa in humans [5], [6], wildlife [5], [7], mosquitoes [8], [9], [10] and livestock [11], [12], [13]. Yet, Wesselsbron disease may be an under-recognized cause of abortion, stillbirths, congenital abnormalities, and high neonatal mortality in small ruminants [4], [13], [14] because it closely resembles other common pathogens associated to abortions, including Rift Valley fever, bluetongue, or brucellosis, and is not routinely diagnosed. Humans infected with WSLV typically develop fever, rash, myalgia and arthralgia, with signs of liver involvement [5], [6], [15], [16], [17]; serological surveys indicate exposure across many African countries [6], [18].

Recent findings underscore the need to consider WSLV from a One Health perspective [19]. A severe outbreak in dromedary camel calves in the Borana Zone of Ethiopia confirmed systemic WSLV infection by molecular diagnostics in a host previously known only from serological surveys [20]. Camel production is expanding across African drylands as recurrent drought has pushed producers away from cattle [21]. Experimental infection of sheep showed that WSLV is transmitted from infected ewes to suckling lambs via milk [22], a previously unrecognized route. This review synthesizes WSLV recognition, ecology, transmission, pathogenesis, diagnosis, immunity, control, and research gaps.

## 2 History and geographical distribution

WSLV was first recognized in 1955 on a sheep farm in South Africa, where it caused high mortality in newborn lambs and abortion in pregnant ewes [4]. Initially mistaken for bluetongue because it recurred in late summer along the Brak River [23], examination of liver and brain from dead lambs established Wesselsbron disease as distinct from bluetongue and Rift Valley fever [24]. Human infection was recognized immediately. Five laboratory workers exposed to outbreak material seroconverted or yielded virus [4], and virus was isolated from a mosquito collection in Tongaland [25]. These seminal cases established WSLV zoonotic potential and direct-contact transmission independently of mosquitoes. Reported human cases are summarized in Table 1.

**Table 1:** Location and context of reported human cases of Wesselsbron disease.

Location (year)	Exposure history	Reference
	Laboratory-acquired infections	
South Africa, Wesselsbron, Free State (1955)	Five veterinary laboratory workers exposed to outbreak isolates; one confirmed by isolation, four by seroconversion	[4]
Uganda, Entebbe (1958)	Laboratory-acquired; encephalitis, transient hearing loss, cognitive impairment	[44]
Senegal, Dakar (1965)	Laboratory-acquired infection	[15]
USA, New Haven (1969)	Laboratory-acquired; possible aerosol transmission	[43]
Nigeria, Ibadan (1972)	Laboratory-acquired infection	[28]
Bangui, Central African Republic (1974)	Laboratory exposure	[15]
Bangui, Central African Republic (1981–1983)	Nine cases, one laboratory-acquired	[15]
	Field entomologic exposure	
South Africa, Tongaland, Natal (now KwaZulu-Natal) (1955)	Mosquito collector; virus isolated from blood (strain SAH177). A second field worker seroconverted without virus isolation	[25]
South Africa, Middelburg, Cape Province (now Eastern Cape, Inxuba Yethemba Municipality, Great Karoo) (1957)	Two field workers with mosquito exposure, one also exposed during sheep necropsy; virus isolated from one, seroconversion in the other	[45]
South Africa, Ndumu, Natal (now KwaZulu-Natal) (1966)	Field worker infected while collecting mosquitoes	[15]
South Africa, Pienaars River, Transvaal (now Limpopo, Northwest border area) (1972)	Mosquito collection	[73]
South Africa, Bultfontein, Free State (1996)	Field scientist infected during mosquito collection	[74]
	Naturally acquired community/agricultural exposure	
South Africa, Schweizer-Reneke, North West (2010)	Isolated from a suspected RVF case; patient farmed sheep, goats, and cattle	[6]
South Africa, Colesberg, Northern Cape (2011)	Isolated from a suspected RVF case; patient farmed predominantly sheep, also cattle	[6]
Senegal, Kédougou (2013)	Two cases confirmed in patients with acute febrile illness	[5]
	Unclear/Undetermined source of infection	
Senegal, Dakar (1983)	Circumstances unclear	[15]
South Africa, Johannesburg, Transvaal (now Gauteng) (1976)	Circumstances not clearly defined	[73]
South Africa, Bloemfontein, Free State (1974)	Source of infection unclear	[73]

The virus has since been recovered from mosquitoes and vertebrate hosts in South Africa, Mozambique, Bostwana, Angola, Namibia, Madagascar, Kenya, Uganda, Mauritania, South Sudan, Cameroon, Central African Republic, Senegal, Zimbabwe, Nigeria, Ethiopia, and Ivory Coast [5], [6], [8], [10], [12], [13], [20], [26], [27], [28], [29]. A single historical report described WSLV in mosquitoes in Thailand in 1966, but no further evidence supports circulation in Asia [30]. Phylogeographic reconstruction places the origin of WSLV in South Africa in the early 19th century, with spread through southern Africa and long-distance movement into western and eastern Africa in the early 21st century [31], [32].

## 3 Taxonomy, genomic diversity, and molecular features

Following revision by the International Committee on Taxonomy of Viruses (ICTV), WSLV is classified in the genus *Orthoflavivirus* (formerly Flavivirus), family *Flaviviridae* [1]. It belongs to the YFV serocomplex, alongside Banzi virus and Uganda S virus [3]; antigenic relatedness underlies cross-reactivity that complicates diagnosis.

The genome is 10,681 nucleotides long, with a single open reading frame flanked by 5′ and 3′ untranslated regions (UTRs). It encodes three structural proteins, capsid (C), precursor membrane (prM), and envelope (E); and seven non-structural proteins (NS1, NS2A, NS2B, NS3, NS4A, NS4B and NS5), plus 2 K between NS4A and NS4B [5]. Structural proteins mediate virion assembly and cell entry; non-structural proteins drive replication, genome synthesis and immune evasion [33].

WSLV present two clades, I and II, [8], [16]; however, since 1997 all isolates were identified as clade I [6]. Genetic diversity is low: 0–1% at the amino acid level and 0–7% at the nucleotide level [16]. Inter-clade recombination has recently been detected, raising the possibility of emerging variants with altered pathogenicity [16]. WSLV appears genetically stable, but recombination and local adaptation may shape its evolution across ecosystems.

The E glycoprotein is the principal surface antigen, mediating receptor binding and membrane fusion and eliciting neutralizing antibodies [34]. Representative clade strains cross-neutralize efficiently [22]. One amino acid substitution in E (V459M) has been associated with abortion and maternal-to-fetal transmission [16] suggesting that E mutations may shift pathogenicity and tissue tropism. However, this remains to be experimentally proven using reverse genetics.

## 4 Ecology, host range, and transmission

### 4.1 Ecology and mosquito vectors

Originating in South Africa in the early 19th century, WSLV expanded into eastern and western Africa in the early 21st century [32] and is now widely distributed across sub-Saharan Africa. Ecological suitability spans semi-arid pastoral rangeland, riverine habitats and wetlands. It is associated with precipitation, urbanization, human and livestock density, particularly in mixed smallholdings where mosquitoes coexist with livestock [32]. Modelling identifies these factors as drivers of northward range expansion under climate change, with risk shifting from rural pastoral to urban and peri-urban zones by mid-century [32]. Outbreaks follow heavy rainfall and rising mosquito numbers, though in the wetter coastal regions of eastern and southern Africa transmission may continue year-round [35], [36].

Most isolates come from *Aedes* species, including *A. circumluteolus*, *A. mcintoshi*, *A. caballus*, *A. tarsalis*, *A. lineatopennis*, *A. mediolineatus,* and *A. minutus* [10], [37], [38]. Several are anthropophilic and may mediate spillover to humans, whereas *Aedes* predominance partly reflects entomological sampling bias [9], *Aedes* species*,* particularly floodwater species, are defined as field vectors. The virus has also been detected in *Mansonia uniformis*, *Anopheles pharoensis*, *Anopheles gambiae*, *Culex telesilla, and Culex univattis* [9], [10]*,* making multiple genera plausible vectors. Vector competence was demonstrated experimentally for *A. aegypti* and *Cx. quinquefasciatus* [39]; however, *Cx. quinquefasciatus* infection required feeding at higher viral titers and had a longer incubation time and lower transmission rate than *A. aegypti* [39]. To date, *Culex* species have not been implicated in field transmission.

### 4.2 Vertebrate hosts

Sheep are the primary recognized vertebrate host and the source of the original isolate [4]. They remain the species most frequently associated with severe disease [4], [22], [40]. WSLV seroprevalence has been determined in livestock including goats, cattle, and camels, and wild animals including buffalo, antelope, wildebeest, zebra, rhinoceros, horses, donkeys, monkeys, birds (toucan) and rodents [5], [7], [41], [42], indicating broad circulation at the wildlife-livestock interface. Although rodents have been proposed as maintenance hosts, no definitive host has been identified [5]. Host roles are summarized in Table 2 and Fig. 1.

**Table 2:** Relative role of different animals in WSLV transmission.

Species	Role	Key findings	References
Sheep	Primary amplifying host	High mortality in newborn lambs; abortion in pregnant ewes; hepatitis in most cases; best-characterized host	[15], [22], [40]
Goats	Amplifying host	Abortion, congenital malformation, and stillbirth reported; systemic infection experimentally	[13], [52]
Cattle	Susceptible host; possible amplifying host	Usually mild or subclinical; occasional transplacental infection; viremia not well characterized	[12]
Camels	Susceptible host; role unclear	Severe systemic disease and high mortality in young animals in a single reported outbreak	[20]
Humans	Presumed incidental (dead-end) host	Usually mild febrile illness; rare hepatic and neurological involvement	[5], [6]
Wild ruminants / rodents	Possible maintenance hosts	Infection detected; role in virus maintenance unknown	[5], [11]
Mosquitoes (*Aedes)*	Vector	Transmit virus between hosts; floodwater *Aedes* incriminated as field vectors essential for WSLV circulation	[9]
Mosquitoes (*Culex*)	Experimentally competent vector	Laboratory competence demonstrated at high oral doses; higher infection threshold, longer extrinsic incubation, lower transmission efficiency at low doses; not implicated in field transmission	[39]

**Fig. 1:**
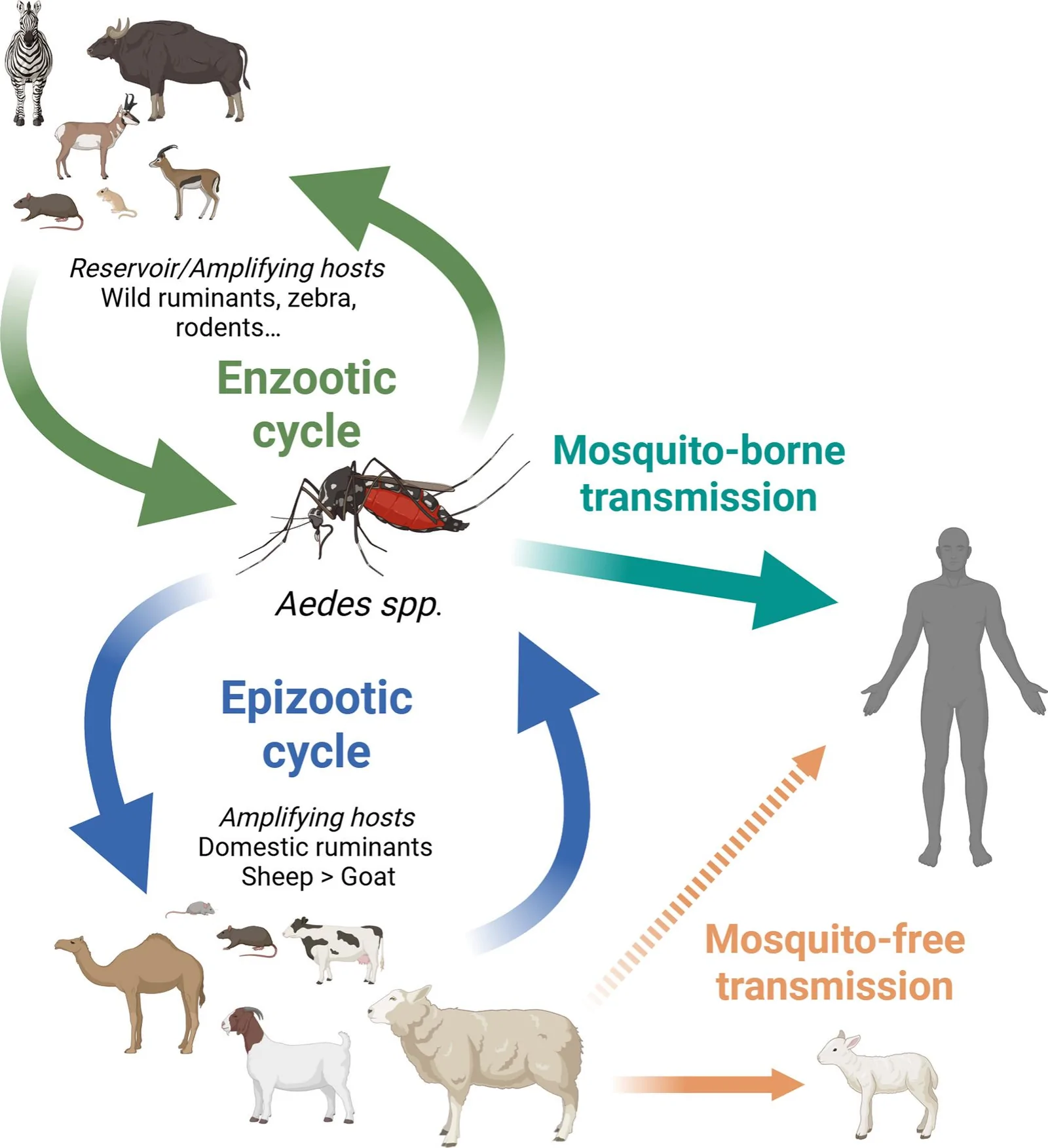
The Wesselsbron virus life cycle. (Created in BioRender. García-Nicolás, O. (2026) https://BioRender.com/hvacjva). WSLV is maintained in an enzootic cycle involving primarily mosquitoes and wildlife reservoirs or amplifying hosts, including wild ruminants. Under favorable ecological conditions, transmission may expand into an epizootic cycle, where domestic ruminants serve as major amplifying hosts. Humans are infected primarily through the bites of infected mosquitoes but may also acquire infection through mosquito-free transmission.

### 4.3 Non-vector transmission

Transmission occurs readily in the absence of mosquitoes. Laboratory-acquired human infections have followed handling of infectious material [4], [43], [44], and cases have been linked to tissue contact during necropsy or consumption of infective carcasses [6], [23], [45], [46]. Although specific mechanisms of direct human transmission remain uncharacterized, WSLV is an occupational and biosafety concern. In sheep, vertical transmission underlies the reproductive losses: following maternal infection the virus crosses the placenta and infects fetal tissue, causing abortion, congenital malformation, stillbirth or neonatal death [4], [14], [20], [40]. Experimental infection demonstrated high WSLV RNA loads in nasal secretions and milk of infected animals and, under insect-free conditions, transmission of clade I and clade II WSLV from lactating ewes to suckling lambs through infectious milk [22].

### 4.4 Livestock production systems and human–animal interfaces

Across much of sub-Saharan Africa, sheep and goats underpin pastoral and agropastoral production, providing meat, milk, household income, and financial security [47]. In arid and semi-arid regions, camels are becoming increasingly important because of their drought resilience [21]. Most livestock are managed extensively on communal grazing lands, with seasonal movements, shared pasture and watering points, and animals kept close to households during lambing and kidding [48], [49]. Small ruminants and camels are also frequently raised in peri-urban and backyard systems, increasing contact between livestock and people.

Husbandry in these settings means repeated, hands-on exposure: milking, assisting parturition, handling aborted fetuses and placentas, nursing sick animals, slaughtering, and conducting post-mortem examinations. Each activity increases WSLV exposure via contact with contaminated tissues and consumption of products from infected animals [6], [22], [40], [43], [45], [46].

Milk deserves particular attention. Raw milk from sheep, goats and camels is widely consumed in pastoral and agropastoral households, and WSLV reaches high infectious titers in milk of experimentally infected ewes, sufficient to infect and cause lethal disease in suckling lambs [22]. The close interface between people, livestock and vectors creates conditions for substantial but unrecognized livestock reproductive loss and occasional zoonotic transmission.

## 5 Clinical disease and pathogenesis

### 5.1 Sheep

Sheep are the most susceptible host, with efficient viral replication in placental and fetal tissues [40]. Mortality in newborn lambs may reach 27% [50]. Hepatitis is the primary pathological manifestation [22], [51]; icterus, hepatic necrosis and degeneration were described in early sheep outbreaks [23]. After a 1–3 day incubation period, lambs may die within 72 h following the appearance of clinical signs [52]. Clinical signs are nonspecific, including fever, anorexia, weakness, rough coat, increased respiratory rate, and depression [11], [22], [50], [53], but icterus and photodynamic dermatitis occur, reflecting disease severity [22], [23], [54].

Pregnant ewes present a transient viremia, then placental infection and fetal disease: congenital neural malformations with arthrogryposis, polyhydramnios, and abortion are common [14], [40]. Viral antigen and lesions have been found in fetal neuronal progenitor cells and differentiated glia, including microglia, astrocytes and oligodendrocytes, demonstrating fetal brain infection after placental crossing [40]. Non-pregnant sheep were long thought to remain subclinical, or to show no more than a brief mild fever [53]. However, recently ewes infected with SAH177 or clade I SA999 strain developed moderate clinical signs, including high fever, labored breathing, reduced milk production, jaundice, and reduced activity [22]. In neonatal lambs, disease severity ranges from mild to severe, reaching about 30% fatality rate. After a short viremia (3–6 days), disease can evolve rapidly with death within days of clinical signs onset. Increased hepatic biochemical parameters (bilirubin, bile acids and AST) can help assess severity; concurrent creatinine elevation indicates severe disease with renal dysfunction [22]. Where biochemical testing is unavailable during outbreaks, monitoring icterus can indicate disease progression and severity. Healthy serum is clear to lightly yellow; progressive jaundice produces increasingly yellow, dark yellow or orange in severe, often fatal cases [22]. While some SAH177-infected animals presented moderate meningoencephalitis lesions, the degree of experimentally reported neurotropism deserves caution. The widely used clade II SAH177 strain was passaged extensively in suckling mouse brains before use, which may have selected for enhanced neurovirulence [22]. Lethal WSLV-associated liver disease in ewes was also described in early reports [23]. Both clades co-circulated in the 1950s without discernable clinical or serological differences; as in recent studies, hepatitis was hallmark [4], [22], [23]. WSLV appears hepatotropic regardless of clade.

A very severe form causes hemorrhages, including bloody nasal discharge and petechiae, and was associated with 100% mortality [23]. Serial complete blood cell count can help monitor progression and severity, as severe thrombocytopenia is associated with coagulation disorders and increased hemorrhage risk. During disease, dehydration and decreased milk production reduce daily weight gain in lambs, including uninfected lambs [22]. No WSLV-specific antiviral exists; care is supportive providing hydration and shelter.

### 5.2 Goats and cattle

Goats are susceptible and develop systemic infection with fever, viremia, and wide tissue dissemination. In Red Sokoto goats infected experimentally with a Nigerian strain, viremia appeared 24–72 h after inoculation and lasted 3–4 days, and virus was detected in liver, spleen, lungs, brain, kidneys, adrenal glands, lymph nodes, and heart [52]. Abortions and congenital malformations occur [13], but their mechanisms are undefined.

Disease is milder in cattle. Experimentally infected animals developed transient fever, short viremia (about 2 days), and severe disease is uncommon [4], [12]. Transplacental infection has been documented by a weak calf that died shortly after birth and precolostral antibodies indicating intrauterine infection, but most calves born to infected cows show no fetal-infection signs [12]. Infection in cattle is therefore predominantly subclinical, and bovine WSLV pathogenesis remains poorly characterized.

### 5.3 Camels

Until recently, the only evidence of WSLV infection in camels was serological [55]. The Ethiopian outbreak investigated in 2024 indicates that camels can develop severe disease with mortality up to 70%. Affected camels were lethargic and dull; they shivered, showed bilateral lacrimation, labored breathing, tremors, recumbency, constipation and oliguria, and died 2–3 days after onset. Necropsy revealed intestinal edema, mucosal congestion, swollen and congested lymph nodes, and hydropericardium, while viral RNA was detected across internal and lymphoid organs, indicating systemic infection [20]. While experimental infection of camels is needed to better characterize tissue tropism and pathogenesis, current evidence expands the assumed host range of WSLV; particularly important as camel populations rise across African drylands [21].

### 5.4 Other species

WSLV was isolated from a Cape short-eared gerbil in southern Africa and, more recently, from a black rat in Senegal supporting the hypothesis that some rodents may serve as wild reservoirs [5]. In experimental animal models, WSLV causes fatal disease in infant mice following intracranial and intraperitoneal inoculation, while adult mice are only susceptible after intracranial inoculation [4]. Intraperitoneal inoculation causes abortion in rabbits and guinea pigs [4]. For seropositive wild species, exposure is all that is known; viral kinetics, tissue tropism and lesion development remain uncharacterized.

### 5.5 Humans

Despite the virus's broad geographical distribution, few human cases of WSLV infections have been reported (Table 1). Most documented infections involve occupational exposure to mosquitoes, livestock, or laboratory material. Disease onset is abrupt and the acute phase is usually moderate and short, with fever (1–3 days), headache, rigors, myalgia, and arthralgia, recovering without evident sequelae [6]. Blurred or impaired vision has been reported, as has one encephalitis case after ocular exposure, causing transient hearing loss, and cognitive impairment [6], [15], [43], [44]. Several cases describe liver disease, including upper-right abdominal pain, tender liver, or increased transaminases during the febrile phase, including in the encephalitis case [6], [44]. Together, these findings suggest limited neuroinvasive capability in humans, whereas hepatotropism appears more prominent, as in sheep.

In a Zimbabwean serosurvey in the 1970s (then Rhodesia), hemagglutination-inhibition (HI) indicated recent WSLV infection in 297 children [46]. Although HI has cross-reactivity limitations, this finding supports WSLV circulation in endemic populations and suggests that infections are underdiagnosed among patients with undifferentiated febrile illness. Febrile patients with icterus should be considered for WSLV testing. Monitoring hepatic biochemical parameters (bilirubin, bile acids, and transaminases) might help assess disease severity and progression. Patients may develop dehydration; oral or intravenous fluid replacement should be included in supportive care. Fever and pain can be managed with paracetamol (acetaminophen), avoiding aspirin or other NSAIDs until dengue or a highly pathogenic WSLV strain has been excluded [56], because thrombocytopenia and haemostatic abnormalities may occur, while NSAIDs can impair platelet function and aggravate bleeding. Clinicians should evaluate for hemorrhages or petechiae. In such cases, complete blood count can help monitor platelets levels.

## 6 Limitations

Much of this evidence rests on a thin foundation typical of neglected diseases, and many aspects must be re-examined. The clinical picture in sheep, goats and cattle derives largely from field observations and experimental infections carried out between the 1950s and 1990s, with few modern studies [12], [22], [23], [40], [50], [52], [53], often using small numbers of animals and without molecular confirmation. Evidence in camels currently comes from a single outbreak report [20]. In experimental settings, the reported neurotropism of clade II SAH177 may be an artefact of extensive passage in newborn mouse brains [22]. Historical seroprevalence literature was built on HI and complement-fixation (CF) assays, which broadly cross-react across the YFV serocomplex. Strain-level variation may also contribute to underdiagnosis; WSLV generally causes liver disease, but highly pathogenic strains may cause peracute disease with more frequent haemorrhagic disease [23], whereas low-pathogenicity strains may cause mild or unapparent infection, as suggested for M5937-UG-2018 [31]. Human cases are predominantly occupational or incidental detections during RVFV-outbreak or febrile-illness investigations (Table 1), reflecting who is tested rather than infected and supporting substantial underdiagnosis.

## 7 Diagnosis, sampling, and immunity

### 7.1 Serological diagnosis

Because Wesselsbron disease symptoms and mosquito vectors closely overlap those of Rift Valley fever (Table 3), laboratory confirmation is essential [24]. Serological tests can clearly differentiate between these viruses. WSLV serological diagnostic methods include virus neutralization tests (VNT), HI, CF, and enzyme-linked immunosorbent assays (ELISA) [4], [12], [57], [58]. The central challenge is cross-reactivity within *Orthoflaviviruses*. CF cross-reacts less than HI in sheep, cattle, and guinea pig sera [12], [58]; while ELISA, run on untreated sera at a single dilution, suits large-scale screening [58], [59]. WSLV-specific exposure requires confirmation by VNT, performed in biosafety level 3 laboratories. Cell culture can replace mice for this purpose [60]; lamb kidney, baby hamster kidney (BHK), and Vero cells are suitable [22], [37], [61].

**Table 3:** WSLV vs RVFV: Clinical and pathological comparison.

Feature	WSLV	RVFV	Other differentials
Abortion and reproductive loss	Common: abortion, stillbirth, congenital malformation, neonatal mortality [4]	Common: abortion storms, fetal death, stillbirth, neonatal mortality [75]	Bluetongue [76], Border virus disease (BDV) [77], Brucellosis [78], Leptospirosis [79], Campylobacteriosis [80], Chlamydiosis, especially *Chlamydia abortus*[81], Salmonellosis [82]
Congenital abnormalities	Well documented, including arthrogryposis and CNS malformation in lambs [14], [50]	Reported less consistently; abortion and fetal death predominate [75]	Akabane virus [83], Aino virus and Schmallenberg viruses [84], Cache Valley virus (CVV) [85], other Simbu-group viruses [86]
Hepatic pathology	Hepatitis is the principal pathological manifestation [22], [23], [51]	Severe hepatic necrosis within systemic disease, especially in young animals [75]	–
Neurological involvement	Fetal CNS lesions after transplacental infection [40]; experimental neurotropism confounded by strain passage history [22]	Encephalitis in both animals and humans [87]	Arboviruses with neurotropism (e.g., Simbu-group) [88], [89]
Human disease	Usually mild, self-limiting febrile illness; rare hepatitis, encephalitis, ocular disease [6]	Mostly mild, but hepatitis, retinitis, encephalitis and haemorrhagic disease occur [87]	Malaria, Dengue, Yellow fever, Chikungunya, other arboviral infections and undifferentiated fevers [90]

### 7.2 Molecular diagnosis

WSLV-specific sensitive fluorescent probe-based reverse transcription-quantitative polymerase chain reaction (RT-qPCR) and reverse-transcription-recombinase polymerase amplification (RT-RPA) assays have been established for E and NS3 genes [16]. While RT-RPA assays are substantially faster, RT-qPCR is 50 times more sensitive and both methods require RNA extraction, limiting RT-RPA utility. Because NS genes, and particularly the E gene, may be subject to evolutionary pressure, a pan-WSLV real-time RT-qPCR assay targeting the conserved 3′-UTR was developed to detect historical and contemporary isolates across genetic diversity [22]. Because serology cannot distinguish WSLV clades, molecular testing, including RT-qPCR or sequencing, is required for clade assignment in human and animal cases.

### 7.3 Sampling strategies

Sample choice should follow pathogenesis. In the acute phase, blood or serum is suitable for molecular detection. But WSLV continues to replicate in the liver after viremia has declined and neutralizing antibodies have risen, making liver a highly reliable specimen [22], [51]. In cases of abortion or neonatal mortality, placental tissue, and fetal liver and brain, are preferable diagnostic samples; spleen, kidney, and tonsils, may also carry viral RNA during systemic infection [20], [22], [40]. Prolonged shedding makes milk and nasal secretions useful, non-invasive specimens in infected animals [22].

### 7.4 Differential diagnosis

WSLV belongs in the differential diagnosis of reproductive outbreaks in endemic regions with abortion storms, stillbirth, congenital malformation, or neonatal mortality. This includes RVFV, bluetongue, border disease/pestivirus infection, and other teratogenic arboviruses (including Akabane, Aino, Schmallenberg, Cache Valley, and related Simbu-group viruses), as well as abortion-inducing bacteria including brucellosis, leptospirosis, campylobacteriosis, chlamydiosis, and salmonellosis (Table 4). Suspicion should rise sharply in neonatal lambs with severe hepatitis and icterus, reflecting marked hepatotropism [22], [51]. WSLV-specific molecular and serological testing should be performed whenever RVFV is suspected, particularly when RVFV testing is negative despite compatible clinical and pathological findings. Testing for both viruses in parallel can improve outbreak attribution and identify WSLV as a primary or concurrent etiological agent.

**Table 4:** Practical guide to sample selection and assay choice for WSLV.

Clinical or epidemiologic context	Samples of choice	Primary Assay(s)	Interpretation/Notes
Acute febrile illness in any host, including humans	Whole blood, serum	RT-qPCR; paired sera for seroconversion; VNT	Viraemia is brief, samples should be collected early. A negative RT-qPCR does not exclude infection [16], [22]
Abortion, stillbirth, or congenital disease	Placenta; fetal liver; fetal brain; whole fetus; Serum	RT-qPCR; VNT	Fetal liver is often the highest-yield tissue, given the hepatic tropism [22], [40], but placental and neural tissues may also be informative
Neonatal mortality in lambs, kids or calves	Liver; also spleen, kidney, tonsil; brain if neurological signs are present	RT-qPCR; histopathology; VNT	Liver is often a key target [22], [51], pancreas, kidney and tonsils should also be sampled. Serum from convalescent animals
Lactating animals	Milk; Nasal secretions, Serum	RT-qPCR; VNT	Milk shedding has been demonstrated in ewes [22]; but this route has not yet been established in goats, cattle and camels
Suspected outbreak with RVFV-negative results	As above, plus paired sera	RT-qPCR for WSLV and RVFV in parallel; VNT for confirmation	HI and CF assays cross-react within the YFV serocomplex; VNT is needed for confirmation [12], [58]
Retrospective investigation/ serosurvey	Serum	ELISA for screening, VNT for confirmation	ELISA alone cannot reliably distinguish WSLV from related *Orthoflaviviruses*[58], [59]

The same logic applies to humans. A patient in an endemic area with fever and contact with small ruminants or camels should be considered for WSLV testing, particularly if differential diagnosis tests were negative for other *Orthoflaviviruses* (such as dengue virus, ZIKV, or YFV), Chikungunya, RVFV and malaria. Recent parturition or abortion in the herd, participation in a necropsy, or occupational contact with livestock tissues should all raise the index of suspicion. Table 4 summarizes samples and assays for common presentations.

### 7.5 Immune responses

The earliest evidence for immune control came from mice, where adults resisted peripheral challenge better than newborns [4], with resistance correlating with macrophage activity in the liver [62]. In experimentally infected sheep and goats, neutralizing antibodies appeared within 5–6 days as viremia fell [22], [52]. Histologically, hepatic immune infiltration was dominated by T cells (approximately 10-fold higher density than B cells), with macrophage/Kupffer cell recruitment showing the strongest correlation with viral load, implicating cell-mediated inflammatory responses in viral control and hepatocyte injury, increasing the transaminases release [51]. While CF and HI antibodies developed in all infected animals, neutralizing antibodies were found predominantly in survivors [52], suggesting that humoral responses contribute to protection. However, fatal hepatitis and continuous WSLV replication in the convalescent livers despite neutralizing antibodies indicate that antibody-mediated clearance alone cannot prevent direct hepatocyte damage and that outcome depends on early immune control and the extent of inflammatory hepatic injury [22]. Recovered animals are thought to develop strong and potentially long-lasting immunity [24].

## 8 Control, prevention, and risk management

Vector control remains the cornerstone of prevention, managing water and breeding habitats, applying larvicides and insecticides, using repellents or insecticide-treated materials in livestock housing, and keeping animals away from heavily infested areas after heavy rain or flooding. Biological approaches developed for other *Orthoflaviviruses* may eventually apply, endosymbiotic *Wolbachia* in *Aedes aegypti* interferes with ZIKV and DENV replication through XRN1-mediated degradation of viral RNA [63], [64], but this approach has not been tested against WSLV.

Vector control alone will not be enough. Human infections have followed laboratory accidents, tissue handling, and necropsy [4], [6], [43], [44], and ewes can transmit WSLV to lambs through milk [22]. Biosecurity, safe necropsy practice, and caution with milk from affected flocks must complement vector management, particularly in the smallholder and pastoral settings.

No vaccine exists. A live attenuated vaccine derived from the original isolate by serial intracerebral passage in mouse pups elicited protective immunity, alone or combined with attenuated RVFV vaccine, but the attenuated virus crossed the placenta, causing fetal malformation and abortion in pregnant animals [65].

Control ultimately depends on a One Health approach linking animal, human and vector surveillance. Clinical overlap with RVFV leads to misattribution of febrile illness, neonatal mortality and reproductive loss. Human cases in Senegal [5] and the Ethiopian camel outbreak [20] highlight under-recognized in livestock and humans.

## 9 Research gaps and priorities

### 9.1 Pathogenesis

What determines tissue tropism, placental infection, and neonatal disease is unknown. The route by which WSLV crosses the placenta, infects fetal tissue, and causes abortion or malformation requires definition. Comparative infection studies in sheep, goats, and cattle would show whether the mechanisms behind abortion, congenital defects and neonatal death are conserved, and why severity differs between species. Whether camels amplify the virus or act as dead-end hosts requires investigations. Development of a rodent model may help accelerate discovery. How viral genetic variation affects tissue tropism and virulence, including the E gene V459M substitution associated with abortion [16], remains to be experimentally demonstrated.

### 9.2 Non-vector transmission

Milk-associated transmission [22] raises further questions: how long ewes shed virus in milk, how WSLV reaches the mammary gland, and how much this route contributes to maintenance and spread. The range of susceptible mammalian cell types has not been mapped systematically and should include epithelial and endothelial cells, immune cells, and parenchymal cells of target organs. Whether raw milk from infected small ruminants or camels can infect people, and how to inactivate the virus in milk, are major knowledge gaps with immediate implications for advice to pastoral households.

### 9.3 Epidemiology

The burden of WSLV is unknown and probably underestimated. The Ethiopian outbreak suggests camels may be clinically important hosts rather than incidental spillover species [20].Their infection frequency, age-related susceptibility, outbreak drivers and role in amplification require definition. More broadly, WSLV contributions to abortion storms, perinatal mortality, and febrile illness, particularly outbreaks first attributed to RVFV, will become clear only if the virus is routinely included in surveillance and differential diagnosis.

### 9.4 Vector competence

Beyond some *Aedes* species endemic to Africa, vector competence is unknown for most mosquito species from which WSLV has been identified. Competence studies should cover African populations and mosquitoes from Europe, Asia, and the Americas, to gauge what would happen if the virus reached a naïve ecosystem.

### 9.5 Diagnostics and vaccines

Serological assays that reliably discriminate WSLV from related *Orthoflaviviruses* are needed, together with wider use of sensitive molecular assays during outbreak investigation; both would help identify reproductive losses currently filed as RVFV-negative. Vaccine development has barely begun, and correlates of protection are undefined. The E protein is the obvious first target, since it mediates attachment and entry and is the principal target of neutralizing antibodies [66]. Low genetic diversity and cross-clade neutralization [22] suggest that prM/E-based vaccines could protect broadly against circulating strains, and recombinant subunit, mRNA, viral-vectored and virus-like particle platforms are candidates. NS1 may serve as a complementary antigen, contributing to the clearance of infected cells even though it is absent from mature virions [67], while the conserved NS3 and NS5 proteins are better suited to antiviral drug development [68], [69]. Remdesivir (Veklury; GS-5734) and Galidesivir (BXC4430) have preclinical activity against *Orthoflaviviruses*, including YFV [70], [71], [72], candidates worth evaluating against WSLV.

## 10 Conclusion

WSLV causes abortion, congenital malformations, neonatal mortality, and systemic disease across several host species, and remains among the most neglected arboviruses in African livestock. Sheep are the best-characterized host and reference model for pathogenesis; the recent camel outbreak shows that severe disease is not confined to small ruminants. Transplacental and milk-borne transmission complicate ecology at the close human-livestock interface of pastoral and smallholder systems. That makes WSLV a One Health problem rather than a solely veterinary problem.

Key recognition clues are hepatitis with icterus in infected sheep, reproductive loss in pregnant animals, and clinical/ecological overlap with Rift Valley fever. Because suspected RVFV outbreaks are routinely investigated without testing for WSLV, its contribution to abortion storms, neonatal mortality, and febrile illness is almost certainly underestimated. Including WSLV in differential diagnosis would establish its true burden. Work on transmission, pathogenesis and vaccines could then make control possible.

## CRediT authorship contribution statement

**Kelly Garina Mugisha:** Writing – original draft, Methodology, Data curation, Conceptualization. **Obdulio García-Nicolás:** Writing – review & editing, Validation, Data curation, Conceptualization. **Moses M. Njire:** Writing – review & editing. **Cecilia R. Waruhiu:** Writing – review & editing. **Charaf Benarafa:** Writing – review & editing, Validation, Supervision, Funding acquisition. **Eric M. Fèvre:** Writing – review & editing, Validation, Supervision, Funding acquisition.

## Funding

This work was funded in part by a grant of Wellcome Trust, grant reference 319565/Z/24/Z, the Multidisciplinary Center for Infectious Diseases (MCID) grant: MA_17 and the 10.13039/501100001711Swiss National Science Foundation (233577 to CB).

## Declaration of competing interest

The authors declare that they have no known competing financial interests or personal relationships that could have appeared to influence the work reported in this paper.

## Data Availability

All the data used in this review paper are previously published data which are available online.
